# Supplementary material for: A National Survey of Dispensing Practice and Customer Knowledge on Antibiotic Use in Vietnam and the Implications
Source: Antibiotics (Basel). 2022 Aug 12;11(8):1091. doi: 10.3390/antibiotics11081091 (PMC9405246; doi:10.3390/antibiotics11081091)
Supplement: Supplementary file 1 [file antibiotics-11-01091-s001.zip › antibiotics-1836198-supplementary.pdf]

CODE:

STUDY QUESTIONNAIRE (Translated)

Province/City:	District:	Commune:	Address:
Name of drug retail:<br>☐Pharmacy: ……………………..☐Drugstore:………………………			Date of survey<br>…../…../………..

PART 1. ANTIBIOTIC PURCHASING BEHAVIOR						
1.1.	Does customer buy **drugs** with or without prescription?	☐ With prescription ☐ Without prescription				
1.2	Does customer buy **antibiotics**?	☐ Yes ☐ No				
1.2.1	If yes, with or without prescription?	☐ With prescription ☐ Without prescription				
1.2.2	In case of buying antibiotics **without prescription**, the customer	☐ Request for specific antibiotics<br>☐ Describe illness/symptoms				
1.3	How many types of antibiotics are purchased?	…………………………………………………………				
	**Antibiotics purchased**	**1**	**2**	**3**	**4**	**5**
1.4.	Drug name - Dosage					
1.5.	Active ingredients					
1.5.1	ATC Code					
15.2.	AWaRE Classification					
1.6	Units *(tablets, vials, ampoules, ...)*					
1.7	Amount					
	**PART 2. ANTIBIOTIC KNOWLEDGE**					
2.1	For what illness is this antibiotic?..					
2.2	According to you, it is illegal for purchasing of antibiotics without a doctor's prescription?<br>☐ Yes ☐ No ☐ Unknown					
2.3	In the future, do you agree to visit the doctor for having a prescription when purchasing antibiotics at the pharmacy/drugstore?<br>☐ Agree ☐ Partly agree ☐ Disagree ☐ Not sure					
2.4	According to you, normally, how long should antibiotics be taken?<br>☐Only for a short time (e.g. 1-2 days), stop using if feeling better<br>☐At least five-seven days even if feeling better<br>☐Unknown<br>☐Other (*please specify*): ……………………					

2.5	According to you, does not taking antibiotics for long enough lead to antibiotic resistance?<br>☐ Yes ☐ No ☐ Unknown
2.6	Have you ever known/heard about "antibiotic resistance"?<br>☐ Unknown ☐ Yes, *please specify*…………………<br>*If yes, from what source?*<br>☐Doctor ☐Drug seller ☐Television, Radio, Newspaper ☐Internet ☐Other: ………………
2.7	Is antibiotic resistance a serious problem in the community?<br>☐ Agree ☐ Partly agree ☐ Disagree ☐ Unknown
2.8	*If a customer buys antibiotics without a prescription, why do you think they go to the pharmacy/drugstore instead of visiting the doctor to get a prescription?*<br>☐Disease or symptoms are mild, not serious<br>☐Saving time compared to visiting the doctor<br>☐Saving money compared to visiting the doctor<br>☐Having personal experience with successfully treating these conditions<br>☐Personnel in the drugstore/pharmacy often treats these diseases or symptoms many times beforehand<br>☐Other (*please specify*): …………………………………………….
	**PART 3. PARTICIPANTS' DEMOGRAPHIC CHARACTERISTICS**
	1. Address:...<br>2. Age: …………<br>3. Gender: ☐ Male ☐ Female<br>4. Educational level: ☐ ≤ Graduated high school ☐College ☐University<br>5. Occupation: ☐Freelance work ☐Others (*please specify*):………………………<br>☐Office Staff ☐ Medical Staff
